# Training the equine respiratory muscles: Inspiratory muscle strength

**DOI:** 10.1111/evj.13606

**Published:** 2022-07-07

**Authors:** Laura E. Fitzharris, Melanie J. Hezzell, Alison K. McConnell, Kate J. Allen

**Affiliations:** ^1^ Bristol Veterinary School University of Bristol Langford Bristol UK; ^2^ Independent Consultant Bournemouth Dorset UK

## Abstract

**Background:**

Little is known about the response of the equine respiratory muscles to training.

**Objectives:**

To measure an index of inspiratory muscle strength (IMSi) before and after a period of conventional exercise training (*phase 1*) and inspiratory muscle training (IMT), comparing high‐load (treatment) and low‐load (control) groups (*phase 2*).

**Study design:**

Prospective randomised controlled trial.

**Methods:**

*Phase 1*: Twenty National Hunt Thoroughbred racehorses performed an inspiratory muscle strength test (IMST) twice on two occasions; when unfit at timepoint A (July), and when race fit at timepoint B (October). *Phase 2*: Thirty‐five Thoroughbred racehorses at race fitness were randomly assigned into a high‐load (treatment, *n* = 20) or low‐load (control, *n* = 15) IMT group. The high‐load group followed an IMT protocol that gradually increased the inspiratory pressure applied every 4 days. The low‐load group underwent sham IMT with a low training load. The IMT was performed 5 days/week for 10 weeks. The IMST was performed twice on two occasions, timepoint B (October) and timepoint C (January). Conventional exercise training and racing continued during the study period. The peak IMSi values obtained from the different groups at timepoints A, B and C were compared using a Wilcoxon Signed Rank Test.

**Results:**

*Phase 1*: There was a significant increase in IMSi from timepoint A: 22.5 cmH_2_O (21–25) to timepoint B: 26 cmH_2_O (24–30) (*p =* 0.015). *Phase 2*: From timepoint B to C there was a significant increase in IMSi for the high‐load group 34 cmH_2_O (28–36) (*p =* 0.001) but not the low‐load group 26 cmH_2_O (24–30) (*p =* 0.929). The peak IMSi at timepoint C was significantly higher for the high‐load than low‐load group (*p =* 0.019).

**Main limitations:**

Single centre study with only National Hunt horses undergoing race‐training included.

**Conclusions:**

In horses undergoing race training there is a significant increase in IMSi in response to conventional exercise training and high‐load IMT.

## 1 INTRODUCTION

In human athletes there is a training‐induced adaptation of the respiratory muscles^1^, ^2^ with an increase in the inspiratory muscle strength following both non‐specific strength training and specific inspiratory muscle training (IMT).^1^, ^2^, ^3^ IMT has been used as an ergogenic aid in healthy human subjects, with investigations demonstrating an improvement in athletic performance^4^ and a change in a range of physiological parameters^5^, ^6^ but most importantly attenuation of inspiratory muscle fatigue.^7^ In addition, there is a correlation between diaphragm thickness and inspiratory muscle strength in people,^8^, ^9^, ^10^ with an increase in diaphragm thickness and inspiratory muscle strength (measured by maximal inspiratory pressure) following IMT.^8^, ^10^

Currently little is known about the response of the equine respiratory muscles to training. Methods to directly assess muscles of the equine respiratory system include ultrasound measurement of muscle size^11^, ^12^, ^13^ and electromyographic measurement of function.^14^, ^15^ Recently, a method to measure an index of inspiratory muscle strength (IMSi) has been developed whereby the horse undertakes an incremental inspiratory loading protocol until failure to tolerate the load occurs.^16^ In addition, equipment and protocols for the application of IMT in horses have been developed.^16^ Preliminary investigations conducted in racehorses have shown good feasibility and demonstrated that following a period of IMT there was an increased in IMSi in horses both at rest^17^ and undergoing race training,^16^ counteracting the loss of strength associated with detraining.^17^ The study reported here was conducted as part of a larger body of research investigating the response of the equine respiratory muscles to training.^18^

This study was composed of two phases; the aim of phase one was to measure and compare the inspiratory muscle strength of unfit National Hunt horses before and after a period of conventional exercise training. The aim of phase two was to measure and compare the inspiratory muscle strength, in horses considered to be race fit and undergoing either a high‐load (treatment) or low‐load (control) IMT program. Our hypothesis was that there would be an increase in inspiratory muscle strength in response to conventional exercise training, with a further increase in response to high‐load IMT. A further objective was to assess the behavioural response during the application of IMT, with the hypothesis that the display of a behavioural response would be higher in horses undergoing high‐load IMT.

## 2 MATERIALS AND METHODS

### 2.1 Phase 1

#### 2.1.1 Population

Thoroughbred racehorses were recruited from a single National Hunt trainer. Inclusion criteria required horses to be considered ‘unfit’ but healthy and not to have undergone any routine exercise for a minimum of 8 weeks before recruitment, to allow a period of detraining from the previous season while maximising case recruitment.^19^, ^20^ Horses were to enter a standard exercise programme to train for competition in National Hunt races.

#### 2.1.2 Inspiratory muscle strength test

All examinations took place in the horse's stable and were performed without sedation. Each horse undertook the inspiratory muscle strength test (IMST) twice on two occasions, firstly at timepoint A when considered ‘unfit’ in July, and again at timepoint B when considered ‘race fit’ 12 weeks later in October.

##### Equipment

A bespoke equine mask has previously been developed to allow the measurement of inspiratory muscle strength in horses, using the electronic POWERbreathe K5® valve (POWERbreathe International Ltd.).^16^ Briefly, the mask is composed of plastic that covers the entire muzzle, with latex rubber forming an airtight seal, secured in place with a Velcro® fastened head piece. An opening at the level of the nares allows the attachment of the electronic POWERbreathe K5® valve, which applied the IMST protocol.

##### Acclimatisation

The horses were introduced to wearing a loose‐fitting mask with large air holes during the first session. Gradually, a training mask and low‐load (5 and 10 cmH_2_O) Intersurgical® (C‐PEEP™, Intersurgical Ltd.) valves were introduced by an experienced user (LF). The acclimatisation session continued until the horse took three consecutive breaths (full inspiration and expiration) at a normal respiratory rate with each of the two valves. Two acclimatisation sessions were completed before the IMST was undertaken.^18^

##### Inspiratory muscle strength test protocol

**FIGURE 1:**
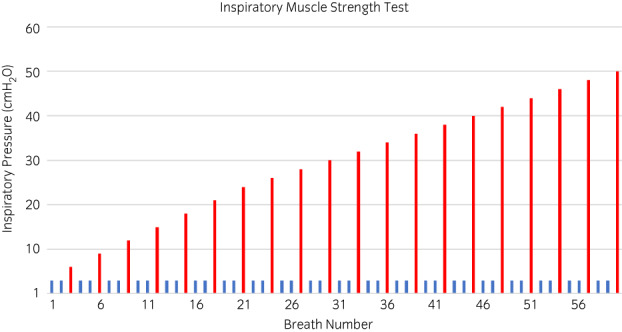
Inspiratory muscle strength test protocol, consisted of a maximum of 60 breaths, alternating between two ‘minimally’ loaded breaths at 3 cmH_2_O and a single ‘loaded’ breath which incrementally increased by 3 cmH_2_O each time up to 24 cmH_2_O, following which the incremental increase was 2 cmH_2_O each loaded breath up to a maximum value of 50 cmH_2_O if all 60 breaths were completed. The red bars represent the loaded breaths and the blue bars represent the minimally loaded breaths.

The IMST protocol consisted of a maximum of 60 breaths, alternating between two ‘minimally’ loaded breaths at 3 cmH_2_O and a single ‘loaded’ breath which incrementally increased by 3 cmH_2_O each time up to 24 cmH_2_O, following which the incremental increase was 2 cmH_2_O each loaded breath up to a maximum value of 50 cmH_2_O if all 60 breaths were completed (Figure 1). The IMST was concluded when the horse was unable to open the load applied by the electronic POWERbreathe K5® valve, despite inspiratory effort on two consecutive attempts, or all 60 breaths were completed. The IMSi was the highest load (cmH_2_O) at which the horse was able to open the valve and was considered the primary outcome measure of interest for assessing inspiratory muscle strength.

##### Examination

The IMST was conducted twice at each timepoint, a minimum of 24 h apart.^18^ The data from the IMST with the highest IMSi was used for the analysis, regardless of whether these were obtained from the first or second IMST at each timepoint.

#### 2.1.3 Exercise training

All horses underwent conventional exercise training, following individual programmes as prescribed by the trainer. The overall aim was to increase fitness between the two examinations at timepoints A and B. All horses were deemed by the trainer to be at race fitness at timepoint B. Information on exercise training was obtained from a telephone questionnaire.

### 2.2 Phase 2

#### 2.2.1 Population

Sample size calculations were performed using GLIMMPSE software (Version 2, https://v2.glimmpse.samplesizeshop.org/#/) based on preliminary data^18^ estimating a 40% change in IMSi in response to IMT with a 15% drop out rate. Based on this information, with power *β* = 0.8 and type 1 error *α* = 0.05, a minimum of 10 horses were to be recruited. As this investigation was conducted alongside ultrasound measurement of the respiratory muscles, which required a larger sample size, additional horses were recruited.^1^ Inclusion criteria required horses to be considered ‘race fit’ by the trainer and to continue in training and competition in National Hunt races for the 10 weeks of the study period.

#### 2.2.2 Inspiratory muscle strength test

Each horse was examined on two occasions, at timepoint B in October, and 10 weeks later at timepoint C in January. The equipment, acclimatisation and IMST protocol were the same as described in phase 1.

#### 2.2.3 Inspiratory muscle training

The IMT was applied using a bespoke mask and inspiratory pressure threshold valves,^1^, ^16^ the IMT protocol has been reported by our group previously.^1^ Briefly, the horses were randomly assigned, into either a high‐load (treatment) group or low‐load (control) group. The high‐load IMT protocol gradually increased the inspiratory pressure applied every 4 days (5, 10, 12.5, 15, 20 cmH_2_O using Intersurgical® valves then in increments of 2.5 cmH_2_O using a POWERbreathe® valve: Medic Classic, POWERbreathe International Ltd.) (Table S1). The low‐load IMT protocol was a low inspiratory pressure of 2.5 cmH_2_O with an extra 5 breaths of a greater load every 5 days, gradually increasing to a maximum of 20 cmH_2_O (5, 10, 12.5, 15, 20 cmH_2_O) (Table S1) to acclimatise the horse to opening a valve with a higher pressure without having a training effect on the muscles. For both groups, the IMT involved two sessions of 3 min duration performed back‐to‐back with a short break in between, undertaken 5 days per week over a period of 10 weeks. A minimum of 40 IMT sessions were to be completed before repeat examination; IMT was not performed in the 48 h prior to racing.

The IMT was performed by either the primary author (Laura E. Fitzharris) or by a research assistant, who had undergone specific training on how to use and apply the IMT correctly.^1^ A training diary was provided for each horse and contained the training protocol stating the inspiratory pressure level to be applied during each training session, with additional notes recorded such as assessment of horse behaviour and equipment failure (Table S1). The trainer and stable staff were blinded as to which horses were in the high‐ and low‐load groups.

#### 2.2.4 Behaviour analysis

For the purpose of this investigation, the term ‘behaviour’ is used to describe any purposeful activity performed/exhibited/displayed by the horse, outside of standing still, during the application of IMT. Following each IMT session, a training diary was completed for each horse, documenting the inspiratory pressure of the IMT performed, whether the IMT was performed without stopping and what behaviours, if any, were displayed by the horse during the IMT session. Retrospective analysis of the training diaries was undertaken to tally the event of different behaviours. An ethogram was composed, detailing the different behaviours observed (Table S2).

#### 2.2.5 Exercise training

All horses were considered at race fitness by the trainer and were to continue conventional exercise training, following individual programmes as prescribed by the trainer, between the two examinations at timepoints B and C.

### 2.3 Data analysis

Data were recorded in Microsoft Excel® and SPSS® (version 24) was used for statistical analysis. The data extracted from the BREATHELINK™ software (POWERbreathe International Ltd.) for each breath taken during each IMST included IMSi (cmH_2_O), average load (cmH_2_O), breath volume (L), peak flow (L/s), peak power (W) and work (J). In addition, the peak values for each of these parameters, obtained at any point, during each IMST were recorded. Data were assessed for normality graphically with histogram plots and a Shapiro–Wilk test. Results are presented as mean (±SD) or median (interquartile range). *Phase 1*: The peak values obtained from the IMST at timepoints A and B were compared using a Wilcoxon Signed Rank Test (IMSi, flow, work, and volume) or a paired samples *t*‐test (average load, and power). *Phase 2*: An independent samples *t*‐test was performed to compare the randomly assigned high‐ and low‐load groups, assessing age, weight, performance ratings [Official Rating (OR), Racing Post Rating (RPR), and Timeform (TF)] and IMSi at timepoint B. The peak values obtained at timepoints B and C, for the high‐ and low‐load groups were compared using a Wilcoxon Signed Rank Test (IMSi, flow, power, work, and volume) or a paired samples *t*‐test (average load). The peak values obtained by the high‐ and low‐load groups were compared at timepoints B and C using a Mann–Whitney *U* Test (IMSi, flow, power, work, and volume) or an independent samples *t*‐test (average load). The different types of behaviours recorded were extracted from individual training diaries, along with the number of occasions that each behaviour was exhibited by each horse. The number of sessions where IMT could not be completed due to the display of avoidance behaviours was recorded. An independent samples *t*‐test was performed to compare the number of sessions where a behavioural response to IMT were recorded between the high‐ and low‐load groups and the odds ratio (OR) calculated. Significance was set at *p* < 0.05.

Trainers gave consent for their animals' inclusion in the study.

## 3 RESULTS

### 3.1 Phase 1

#### 3.1.1 Population

Twenty‐three Thoroughbred racehorses were initially recruited. Three horses did not perform the IMST due to inadequate acclimatisation during the initial two sessions; one horse was unsettled by the noise made by the flow of air through the respiratory valves and did not improve during repeated acclimatisation sessions. The other two horses were both head shy, young (3 years old), large (>600 kg), and new to the yard; attempts to safely apply the training mask were unsuccessful. Overall, 20 Thoroughbred horses performed the IMST (one mare and 19 geldings) with a mean [±SD] age of 6.1 [±1.8] years, and weight of 548.8 [±44.4] kg. All 20 horses were naïve to the IMST equipment and procedure.

#### 3.1.2 Inspiratory muscle strength test

All 20 horses successfully completed the IMST twice at timepoint A and timepoint B.

##### Peak values

**FIGURE 2:**
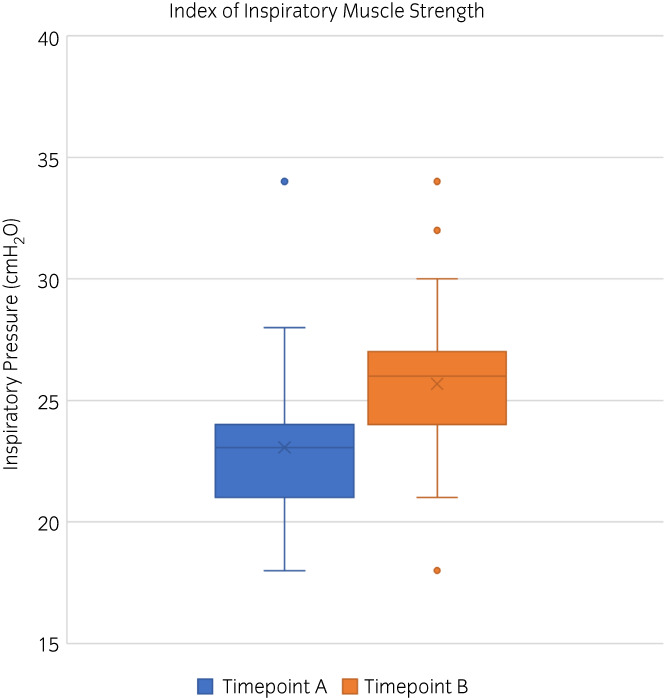
Box and whisker plots for the index of inspiratory muscle strength which showed a significant increase from timepoint A (blue) to timepoint B (orange). *p < 0.05

There was a significant increase in IMSi from timepoint A: 22.5 cmH_2_O^21^, ^22^, ^23^, ^24^, ^25^ to timepoint B: 26 cmH_2_O (24–30) (*p =* 0.02) (Figure 2). In addition, there was a significant increase in the average load (*p =* 0.008), work (*p =* 0.03) and volume (*p =* 0.01) from timepoint A to timepoint B. There were no significant differences in peak flow (*p =* 0.9) or power (*p =* 0.3) between the two timepoints. These results are provided in Table 1.

**TABLE 1:** Results of the inspiratory muscle strength test performed at timepoint a and timepoint B

	Timepoint A	Timepoint B	*p* Value
IMSi (cmH_2_O)	22.5 (21–25)	26 (24–30)	0.02
Average load (cmH_2_O)	24.0 [±3.9]	26.3 [±4.0]	0.008
Power (W)	6.0 [±2.1]	6.4 [±1.9]	0.3
Work (J)	3.8 (3.1–5.0)	4.6 (3.7–6.3)	0.03
Flow (L/s)	4.5 (4.3–4.9)	4.7 (4.4–5.1)	0.9
Volume (L)	6.1 (5.6–6.8)	7.7 (6.8–8.6)	0.01

*Note*: Results are presented as mean [±SD] or median (interquartile range).

#### 3.1.3 Exercise training

Between timepoints A and B walk and trot was undertaken for 10 days to 3 weeks prior to starting canter exercise. The duration and intensity of canter exercise was gradually increased over the following 9‐ to 10‐week period. Canter exercise was performed on either a deep sand circular gallop or hill gallop, with fast work undertaken two or three times per week. Individual training programmes were tailored to each horse, and were dependent on the horse's temperament, experience, locomotor and respiratory ‘health’, fitness level and competition schedule. Additional horse walker exercise was used regularly (5–7 days/week).

### 3.2 Phase 2

#### 3.2.1 Population

The 20 horses that undertook phase 1 were included for phase 2. In addition, a further 20 horses were recruited however, five horses were lost just before the study commenced (five horses were removed from training due to orthopaedic injuries unrelated to the study and therefore withdrawn). No further horses were available that fitted the inclusion criteria. Thirty‐five horses undertook the IMST at timepoint B and started the IMT (one mare and 34 geldings) with a mean [±SD] age of 6.0 [±1.7] years and weight 500.9 kg [±37.8]. Additional sample size calculations were performed with the outcome of having uneven group sizes, with 20 horses in the high‐load treatment group and 15 horses in the low‐load control group. There was no significant difference between the high‐ and low‐load groups at timepoint B in terms of age (*p =* 0.3), weight (*p >* 0.9), performance ratings [OR (*p =* 0.3), RPR (*p =* 0.5) and TF (*p =* 0.4)] or the IMSi (*p =* 0.8) (Table 2).

**TABLE 2:** Comparison of the high‐load and low‐load groups at timepoint B

	High‐load	Low‐load	*p* Value
Age (years)	5.8 [±1.3]	6.4 [±2.1]	0.3
Weight (kg)	500.8 [±37.0]	501.1 [±40.2]	>0.9
Official rating	121.9 [±22.2]	129.4 [±10.3]	0.3
Timeform rating	103.1 [±25.5]	104.7 [±13.8]	0.4
Racing post rating	104.9 [±28.4]	111.9 [±25.6]	0.5
Index of inspiratory muscle strength (cmH_2_O)	26 (24–30)	26 (24–30)	0.8

*Note*: Results are presented as mean [±SD] or median (interquartile range).

Twenty‐eight horses completed the IMT (high‐load = 16, low‐load = 12) and the IMST at timepoint C. Seven horses were lost during the study period: four horses were moved into isolation relating to biosecurity protocols, two horses were removed from training due to orthopaedic injury and therefore IMT was discontinued; one horse developed avoidance behaviours during IMT such that the IMST was not performed at timepoint C.

#### 3.2.2 Inspiratory muscle training

The mean number of days training for the high‐load group was 40.6 [±1.2] days and the low‐load group was 39.5 [±1.5] days. The mean peak training load in the high‐load group was 34.8 [±1.1] cmH_2_O. The low‐load group undertook five breaths of a higher load on seven occasions each.

##### Behaviour

Twenty‐nine horses completed the IMT. Eleven different behaviours were exhibited during IMT (Table 3). All but one horse displayed a behavioural response on at least one occasion. Overall, 837/1087 (77%) of IMT sessions were performed without a behavioural response recorded. Where a behavioural response occurred, for the most part it was infrequently displayed during the session therefore the overall prevalence was low. There was no significant difference in the frequency with which these behavioural responses were displayed between the high‐load group (145/609 [23.8%] training sessions) and the low‐load group (103/478 [21.5%] training sessions) (*p =* 0.4), OR = 1.14 (95% confidence interval = 0.86–1.52). The IMT was successfully completed during every session in 27 horses. Two horses displayed avoidance behaviours such that IMT could not be performed in 11/41 (27%) and 4/43 (9%) of sessions. The behaviours displayed reflected the horses' temperaments during ridden exercise. One horse did not complete the IMST at timepoint C and was removed from the final analysis.

**TABLE 3:** Information obtained from the training diaries on the different behaviours exhibited by horses during inspiratory muscle training, the number of horses that displayed the behaviour and frequency

Behaviour	Number of horses that displayed behaviour	Number ofsessions behaviour recorded	Frequency (%)
Grinding teeth/chewing	12	55	4.7
Head tossing	17	164^a^	14.1
Snorting	2	21	1.8
Wiggling the muzzle	6	17	1.5
Opening the mouth/crossing the jaw	6	63^b^	5.4
Elevating forelimb	2	8	0.7
Low head carriage	2	26	2.2
Yawning	2	4	0.3
Lip smacking	2	5	0.4
Extending the neck	2	7	0.7
Spinning around	1	13	1.1

^a^ One horse displayed behaviour in 39 sessions.

^b^ One horse displayed behaviour in 25 sessions.

##### Equipment

The latex rubber seal ripped during application of the mask and required replacement in four masks. In addition, air leakage during inspiration was reported at the safety clip attachment site of the headpiece, which required the application of extra sealant in six masks. Finally, replacement pressure threshold valves were required for six horses (four low‐load Intersurgical® valves and two high‐load POWERbreathe® Medic Classic valves). Spare masks and valves were available for immediate replacement, so the application of IMT was not affected by these malfunctions.

#### 3.2.3 Inspiratory muscle strength test

##### Population

There were no significant differences in the peak values at timepoint B between the high‐load and low‐load horses for any of the parameters examined: IMSi (*p =* 0.8), average load (*p =* 0.7), power (*p =* 0.8), flow (*p =* 0.4), work (*p =* 0.7) or volume *(p =* 0.6), indicating that the two groups were equal at the start of the IMT (Table 4).

**TABLE 4:** Results of the inspiratory muscle strength test performed by the high‐load and low‐load groups, comparing the results obtained at timepoint B and timepoint C

	High‐load			Low‐load			High‐load versus low‐load	
	Timepoint B	Timepoint C	*p* Value	Timepoint B	Timepoint C	*p* Value	Timepoint B *p* value	Timepoint C *p* value
IMSi (cmH_2_O)	26 (24–30)	34 (28–36)	0.001	26 (24–30)	26 (24–30)	0.9	0.8	0.02
Average load (cmH_2_O)	26.0 [±4.3]	33.8 [±5.2]	<0.001	27.8 [±3.5]	27.3 [±4.4]	0.6	0.7	0.01
Power (W)	5.9 (4.6–7.3)	9.7 (8.4–10.8)	0.001	6.6 (5.8–7.8)	6.0 (4.7–6.9)	0.7	0.8	0.01
Work (J)	5.7 (4.3–7.4)	9.4 (6.1–9.9)	0.02	5.7 (4.4–6.4)	6.4 (4.4–9.5)	0.1	0. 7	0.3
Flow (L/s)	4.7 (4.4–5.1)	5.5 (5.1–6.0)	0.09	4.6 (4.3–5.0)	4.6 (4.1–5.0)	0.8	0.4	0.03
Volume (L)	7.8 (6.9–8.7)	9.8 (8.2–11.1)	0.001	8.1 (7.3–9.8)	8.5 (7.4–10.1)	0.3	0.6	0.3

*Note*: Results are presented as mean [±SD] or median (interquartile range).

##### Peak values

**FIGURE 3:**
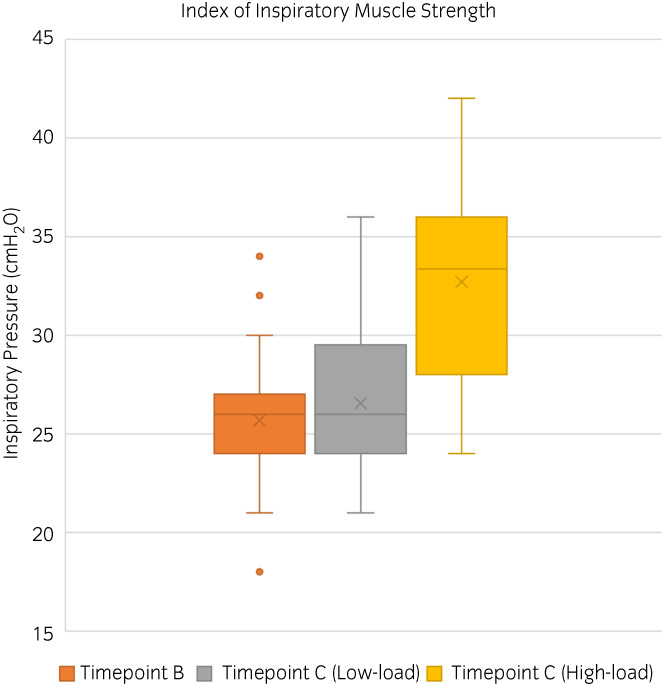
Box and whisker plots for the index of inspiratory muscle strength obtained at timepoint B (orange) and timepoint C for the high‐load (yellow) and low‐load (grey) horses. There was a significant increase for the high‐load group at timepoint C, but not for the low‐load group. *p < 0.05

For the high‐load group there was a significant increase in the peak IMSi from timepoint B: 26 cmH_2_O (24–30) to timepoint C: 34 cmH_2_O (28–36) (*p* = 0.001). In addition, there was a significant increase in the peak values for average load (*p* < 0.001), power (*p* = 0.001), work (*p* = 0.02) and volume (*p* = 0.01), obtained at timepoint C compared to timepoint B (Figure 3). There was no significant difference in flow (*p =* 0.09) (Table 4).

For the low‐load group there was no significant difference in the peak IMSi from timepoint B: 26 cmH_2_O (24–30) to timepoint C: 26 cmH_2_O (24–30) (*p >* 0.9). In addition, there were no significant differences in average load (*p =* 0.9), power (*p =* 0.7), flow (*p =* 0.8), work (*p =* 0.1) and volume (*p =* 0.3), obtained at timepoint C compared to timepoint B (Figure 3; Table 4). Overall, the low‐level IMT load of 2.5 cmH_2_O was <10% of the median IMSi.

At timepoint C, the peak values obtained for the high‐load group were significantly higher than the values obtained for the low‐load group for IMSi (*p =* 0.02), average load (*p =* 0.01), power (*p =* 0.01) and flow (*p =* 0.03) but not for work (*p =* 0.3) or volume (*p =* 0.3) (Table 4).

#### 3.2.4 Exercise training

During the racing season each horse undertook a consistent training programme from timepoint B to C, with fast work undertaken two or three times per week. There was no change in exercise regimen in the time up to competition, but horses were given a few days of lighter exercise after a race.

## 4 DISCUSSION

This investigation is the first to demonstrate a significant increase in inspiratory muscle strength in Thoroughbred racehorses following 12 weeks of conventional exercise training for National Hunt racing. These results are in‐line with similar investigations conducted in human athletes.^21^ Following a 10‐week period of IMT there was a further significant increase in inspiratory muscle strength in the high‐load (treatment) group, but not in the low‐load (control) group. Alongside this, there were significant increases in the power, work, and volume in the high‐load group.

The low‐load group underwent ‘sham’ IMT at a low inspiratory load of 2.5 cmH_2_O which was <10% of the median IMSi obtained at timepoint C, and therefore unlikely to have a training effect on the inspiratory muscles.^5^, ^8^ An additional five breaths of a greater load was performed every 5 days to acclimatise the horse to opening a valve with a higher pressure, again without having a training effect on the inspiratory muscles. The lack of change in the low‐load control group indicates that the increase in IMSi, and other values in the high‐load group reflect a true increase in inspiratory muscle strength and training effect of the IMT, rather than response to ongoing race training, differences between the groups, or an improved ability to perform the IMST through acclimatisation. In addition, the results of this study compliment a previous investigation by Katz et al., conducted in racehorses not undertaking exercise training, where there was an increase in IMSi with high‐load IMT compared with the control group.^17^

The IMST was successfully performed in 20 horses at timepoint A, 35 horses at timepoint B and 27 horses at timepoint C, with all horses undertaking the IMST twice on each occasion leading to 164 successful IMSTs across the three timepoints. The three horses that did not perform the IMST at timepoint A required more time to allow adequate acclimatisation than was feasible within this study. Safety of both the operators and horse was of utmost importance, so the author elected to not continue with these horses in this situation however, conducting the IMST may have been possible had more time been available.

The use of IMT requires careful consideration, particularly the welfare of the horse while undertaking the procedure. Overall, IMT was deemed safe for the horse and operator. However, a wide range of behaviours were recorded during the application of IMT in this population of Thoroughbred racehorses in training. There were no significant differences in the frequency of behavioural responses displayed between the high‐ and low‐load control groups and the majority (77%) of IMT sessions were conducted without the display of any behavioural response. Where a behavioural response occurred, for the most part it was infrequently displayed during the session therefore the overall prevalence was low, reflecting the findings from the previous investigations.^16^, ^22^ In only two horses was it not possible to complete IMT due to the display of avoidance behaviours. In these individuals, the behavioural responses were similar to those exhibited during ridden exercise and therefore could reflect the horse's personality^23^ or a degree of discomfort or difficulty with the IMT. Overall, the tolerance for the application of IMT equipment and training was excellent however, IMT should be carefully applied by an experienced/informed user with recognition of the behavioural responses displayed by horses to reduce the potential risks and enable the correct application of IMT. The assessment of horse behaviour during IMT, as an indicator of horse experience of the procedure, is crucial to optimise horse welfare overall. Further adaptation of the mask and valve design are required before a commercial product could be made available for safe and correct use by inexperienced users, for example, to prevent inadvertent blocking of the valve port with the horse's muzzle. The users in this study prevented this by positioning the mask appropriately and monitoring the horse to ensure that airflow was always maintained.

The application of IMT is used in human athletes to strengthen the respiratory muscles, delaying the activation of the respiratory muscle metaboreflex,^3^ and optimising athletic performance in sports where diaphragm fatigue is performance limitating.^3^, ^4^, ^24^ In addition, in human subjects, IMT reduces the perception of respiratory and limb effort.^3^, ^24^ The horses' respiratory system is thought to be the limiting factor which determines athletic performance.^25^ Further research is required to confirm whether an increase in diaphragm strength reduces the work of breathing, which may translate to a reduction in the horse's experience of breathlessness during strenuous exercise.^26^ Future investigations may explore whether racehorses experience diaphragmatic fatigue, if the exercise induced metaboreflex occurs, and what role this may play in limiting equine performance.

The purpose of this investigation was to assess the change in inspiratory muscle strength in response to conventional exercise training and IMT. The diaphragm is the main driving force of air into the lungs during inspiration, as such, the IMSi is thought to represent diaphragm strength. The results presented here indicate that both conventional race training and high‐load IMT appear to increase the strength of the equine diaphragm, in line with results in human subjects.^8^, ^10^ A linked investigation also demonstrated an increase in diaphragm thickness, measured ultrasonographically, in association with training.^1^ Although the results of the linked investigation did not show an increase in the size of the muscles of the upper airway with IMT, previous studies have indicated the potential use of IMT for the management of dynamic upper airway obstruction in both equine and human athletes.^22^, ^27^ A larger investigation is required to explore any association between IMSi and athletic performance in horses.^4^

Some of the limitations of a previous investigation^16^ were the lack of a control group, a change in the IMST protocol between timepoints and small number of horses investigated. These limitations were addressed in the current study, with the inclusion of a low‐load control group, the same IMST performed at all three timepoints and a larger number of horses assessed. Therefore, the results presented here offer a robust measurement of inspiratory muscle strength in the horse. However, it should be noted that all horses included in this investigation were undergoing race training for National Hunt racing and further investigation is required to determine the effect of IMT in isolation of whole‐body training.^17^ In addition, this investigation was conducted at a single yard, the race training protocol was not standardised, and horses were not evaluated for the presence of upper or lower airway disease. It is not known whether individual training effects, or upper/lower airway disease may have influenced the IMSi results obtained or the application of IMT. Finally, the association between IMT, IMSi, and race performance were not assessed in this investigation as the number of horses included in the study, and the time between examinations, were both deemed too small to obtain a meaningful difference. This study was performed alongside a linked investigation conducted in a larger population of horses which did explore the association between ultrasound measurement of respiratory muscle size and performance ratings.^1^

## 5 CONCLUSION

In conclusion, the results of this investigation show that the prevalence of behavioural abnormalities during IMT and IMST is low, and that there is an increase in inspiratory muscle strength in response to both conventional race training and high‐load IMT. Further investigation into the association between inspiratory muscle size, such as the diaphragm, and other physiological performance variables are required to help determine the optimal method for training racehorses to achieve peak performance while maximising health and welfare.

## AUTHOR CONTRIBUTIONS

Laura E. Fitzharris executed the study. All authors were involved in the study design, data analysis, preparation and final approval of the manuscript.

## FUNDING INFORMATION

Horserace Betting Levy Board (HBLB; Grant number CS022).

## CONFLICT OF INTEREST

None of the authors has any financial or personal relationship that could inappropriately influence the bias of the content of the paper. Alison K. McConnell has previously developed two commercial devices for IMT in human subjects but no longer has any financial interest in either product; she has also authored two books on the topic.

### PEER REVIEW

The peer review history for this article is available at https://publons.com/publon/10.1111/evj.13606.

## ETHICS STATEMENT

University of Bristol animal welfare and ethical approval board approved the study (VIN/13/027).

## Supporting information


**Table S1** Inspiratory muscle training schedule.

**Table S2** Ethogram detailing the different behaviours observed during inspiratory muscle training.

## Data Availability

The data that support the findings of this study are available from the corresponding author upon reasonable request.
