# Supplementary material for: Training the equine respiratory muscles: Inspiratory muscle strength
Source: Equine Vet J. 2022 Jul 7;55(2):306–14. doi: 10.1111/evj.13606 (PMC10084158; doi:10.1111/evj.13606)
Supplement: Supplementary file 1 — Table S1 Inspiratory muscle training schedule. [file EVJ-55-306-s001.pdf]

Equine Veterinary Journal BEVA

Table S1: Inspiratory muscle training schedule.

IMT – High Load

Phase 1: Intersurgical® Valves

Day	Pressure (cmH$_2$O) (Session 1)	Pressure (cmH$_2$O) Session 2
1	5	5
2	5	10
3	10	10
4	10	10
5	10	12.5
6	10	12.5
7	12.5	12.5
8	12.5	15
9	12.5	15
10	15	15
11	15	15
12	15	20
13	15	20
14	20	20
15	20	20

Phase 2: POWERbreathe® valve

16	20	20
17	20	20
18	20	22.5
19	20	22.5
20	22.5	22.5
21	22.5	22.5
22	22.5	25
23	22.5	25
24	25	25

Day	Pressure (cmH$_2$O) (Session 1)	Pressure (cmH$_2$O) Session 2
25	25	25
26	25	27.5
27	25	27.5
28	27.5	27.5
29	27.5	27.5
30	27.5	30
31	27.5	30
32	30	30
33	30	30
34	30	32.5
35	30	32.5
36	32.5	32.5
37	32.5	32.5
38	32.5	35
39	32.5	35
40	35	35
41	35	35
42	35	37.5
43	35	37.5
44	37.5	37.5
45	37.5	37.5
46	37.5	4
47	37.5	4
48	4	4
49	4	4
50	4	42.5

IMT – Low Load

Day	Resistance (Session 1)	Resistance (Session 2)	Extra (5 breaths)	Day	Resistance (Session 1)	Resistance (Session 2)	Extra (5 breaths)
1	2.5	2.5		26	2.5	2.5	
2	2.5	2.5		27	2.5	2.5	
3	2.5	2.5		28	2.5	2.5	
4	2.5	2.5		29	2.5	2.5	
5	2.5	2.5	10	30	2.5	2.5	20
6	2.5	2.5		31	2.5	2.5	
7	2.5	2.5		32	2.5	2.5	
8	2.5	2.5		33	2.5	2.5	
9	2.5	2.5		34	2.5	2.5	
10	2.5	2.5	12.5	35	2.5	2.5	20
11	2.5	2.5		36	2.5	2.5	
12	2.5	2.5		37	2.5	2.5	
13	2.5	2.5		38	2.5	2.5	
14	2.5	2.5		39	2.5	2.5	
15	2.5	2.5	15	40	2.5	2.5	20
16	2.5	2.5		41	2.5	2.5	
17	2.5	2.5		42	2.5	2.5	
18	2.5	2.5		43	2.5	2.5	
19	2.5	2.5		44	2.5	2.5	
20	2.5	2.5	20	45	2.5	2.5	20
21	2.5	2.5		46	2.5	2.5	
22	2.5	2.5		47	2.5	2.5	
23	2.5	2.5		48	2.5	2.5	
24	2.5	2.5		49	2.5	2.5	
25	2.5	2.5	20	50	2.5	2.5	20
