# Supplementary material for: Training the equine respiratory muscles: Inspiratory muscle strength
Source: Equine Vet J. 2022 Jul 7;55(2):306–14. doi: 10.1111/evj.13606 (PMC10084158; doi:10.1111/evj.13606)
Supplement: Supplementary file 2 — Table S2 Ethogram detailing the different behaviours observed during inspiratory muscle training. [file EVJ-55-306-s002.pdf]

Table S2: Ethogram detailing the different behaviours observed during inspiratory muscle training.

Behaviour	Description of behaviour	Reference
Grinding teeth/chewing	Moving of the jaws back and forth with the jaws clenched, rubbing together the upper and lower teeth	(McDonnell, 2003)
Head tossing	Forcefully throwing the head up, down, or sideways	(Kay and Hall, 2009; von Borstel et al., 2011)
Snorting	Forceful, quick, exhalation	(McDonnell, 2003)
Wiggling the muzzle	Purposeful movement of the top lip/muzzle	
Opening mouth/crossing jaw	Opening of the mouth such that the upper and lower teeth were no longer in contact/movement of the mandible to the side of the maxilla	
Elevating forelimb	Lifting the forelimb off the ground and protracting the limb forwards.	
Low head carriage	Holding the head in a low position such that the muzzle was close to the floor	
Yawning	Wide opening of the mouth	(Fureix et al., 2011; Gorecka-Bruzda et al., 2016)
Lip smacking	Purposeful movement of the top and bottom lips against one another	(Slimington, 1975)
Extending the neck	Extending the head and neck from the standard head position normally observed at rest	(Petsche et al., 1995)

References

Fureix C, Gorecka-Bruzda A, Gautier W, Hausberger M. Cooccurrence of yawning and stereotypic behaviour in horses (Equus Caballus). ISRN Zoology 2011, 2011;1-10.

Gorecka-Bruzda A, Fureix C, Ouvrard A, Bourjade M, Hausberger M. Investigating determinants of yawning in the domestic (Equus caballus) and Przewalski (Equus ferus przewalskii) horses. Sci Nat. 2016;103:1-10.

Kay R, Hall C. The use of a mirror reduces isolation stress in horses being transported by trailer. Applied Animal Behaviour Science 2009;116:237-243.

McDonnell SM. A practical field guide to horse behaviour - the equid ethogram. In: *The Blood Horse*, Hong Kong, China, 2003; 375.

Petsche AM, Derksen FJ, Berney CE, Robinson NE. Effect of head position on upper airway function in exercising horses. Equine Vet J. 1995;27(Suppl 18);18-22.

Slimington DL. Bit patent, In: 4,005,564. 1975

von Borstel UK, Pasing S, Gauly M. Towards a more objective assessment of equine personality using behavioural and physiological observation from performance test training. Applied Animal Behaviour Science 2011;135:277-285.
